# Enhanced benzene vapor adsorption through microwave-assisted fabrication of activated carbon from peanut shells using ZnCl_2_ as an activating agent

**DOI:** 10.1007/s11356-024-32973-z

**Published:** 2024-03-25

**Authors:** Sinan Kutluay, Ömer Şahin, Orhan Baytar

**Affiliations:** 1 Department of Chemical Engineering, Faculty of Chemical and Metallurgical Engineering, Istanbul Technical University, 34469 Maslak, Istanbul Turkey; 2 Department of Chemical Engineering, Faculty of Engineering, Siirt University, 56100 Siirt, Turkey

**Keywords:** Microwave-assisted activated carbon, Benzene adsorption, Experimental design, Isotherms, Kinetics

## Abstract

Herein, microwave-assisted activated carbon (MW-AC) was fabricated from peanut shells using a ZnCl_2_ activator and utilized for the first time to eliminate benzene vapor as a volatile organic compound (VOC). During the MW-AC production process, which involved two steps—microwave treatment and muffle furnace heating—we investigated the effects of various factors and achieved the highest iodine number of 1250 mg/g. This was achieved under optimal operating conditions, which included a 100% impregnation ratio, CO2 as the gas in the microwave environment, a microwave power set at 500 W, a microwave duration of 10 min, an activation temperature of 500 °C and an activation time of 45 min. The structural and morphological properties of the optimized MW-AC were assessed through SEM, FTIR, and BET analysis. The dynamic adsorption process of benzene on the optimized MW-AC adsorbent, which has a significant BET surface area of 1204.90 m^2^/g, was designed using the Box-Behnken approach within the response surface methodology. Under optimal experimental conditions, including a contact duration of 80 min, an inlet concentration of 18 ppm, and a temperature of 26 °C, the maximum adsorption capacity reached was 568.34 mg/g. The experimental data are better described by the pseudo-second-order kinetic model, while it is concluded that the equilibrium data are better described by the Langmuir isotherm model. MW-AC exhibited a reuse efficiency of 86.54% for benzene vapor after five consecutive recycling processes. The motivation of the study highlights the high adsorption capacity and superior reuse efficiency of MW-AC adsorbent with high BET surface area against benzene pollutant. According to our results, the developed MW-AC presents itself as a promising adsorbent candidate for the treatment of VOCs in various industrial applications.

## Introduction

Volatile organic compounds (VOCs) are a diverse group of carbon-based chemicals that easily evaporate into the atmosphere. They arise from various sources, including industrial processes, combustion, paints, cleaning products, and solvents. VOCs have a significant impact on both the environment and human health. They contribute to the formation of ground-level ozone and air pollution, which, in turn, can lead to respiratory problems, headaches, and long-term health issues. The control of VOC emissions is essential for environmental protection and air quality management. Research efforts are focused on understanding the sources, distribution and effects of VOCs, leading to the development of regulations and technologies to reduce their presence in the atmosphere. Scientific studies emphasise the importance of reducing VOC emissions for the benefit of both the environment and human health (Ok & Kutluay 2023, Ece & Kutluay 2022, Zhu et al. 2020). Benzene vapor, a type of VOC, is the gaseous form of the colourless aromatic hydrocarbon benzene. It is released from various industrial processes, vehicle emissions and even cigarette smoke. Even low levels of benzene vapor pose serious health risks, potentially causing leukaemia, anaemia and other blood disorders. Its emissions are strictly regulated, and monitoring and reducing exposure to benzene vapor in the workplace and in the environment is critical to public safety and health. Adsorption, a leading VOC abatement technique, efficiently captures and holds VOCs on solid surfaces, preventing their release. Its adaptability to a wide range of adsorbents and ease of implementation ensure wide application. Methods such as activated carbon filtration excel at removing a range of VOCs due to the large surface area available for adsorption. What's more, it is cost effective and energy efficient, making it an environmentally friendly choice for VOC abatement across all industries (Baytar et al. 2020, Dobre et al. 2014, Ece et al. 2021b, Şahin et al. 2021).

Activated carbon with a specific surface area of 1000 to 3000 m^2^/g includes micropores (pore diameter < 2 nm), mesopores (2 nm < pore diameter < 50 nm), and macropores (pore diameter > 50 nm) (Rashidi and Yusup 2017). Activated carbon has been utilized in various chemical processes such as water purification (Şahin et al. 2016), biogas purification (Santos-Clotas et al. 2019), carbon dioxide (CO_2_) capture (Wawrzyńczak et al. 2019), biodiesel production (Lu et al. 2019), methane reforming (Tan et al. 2019), catalyst support material (Baytar 2018), and VOC adsorption (Batur & Kutluay 2022, Baytar et al. 2020; Kutluay et al. 2019a) due to its porous structure and high surface area. Activated carbon can be obtained from wood (Bashkova & Bandosz 2009), coal (Batur et al. 2021), polymer residues (Wei et al. 2012), and agricultural waste (Genli et al. 2022). Currently, there is a growing trend towards the production of activated carbon from biomass and agricultural waste, which are cheap and abundant raw materials (Gong et al. 2013). Therefore, in recent years, researchers have utilized agricultural residues such as palm shells (Adinata et al. 2007), Elaeagnus angustifolia seeds (Kutluay et al. 2019a), cherry seeds (Nowicki et al. 2015), apricot seeds (Soleimani & Kaghazchi 2008), walnut shells (Zabihi et al. 2010), and chickpea stems (Genli et al. 2022) in the preparation of activated carbon. There are two fundamental steps in the production of activated carbon: carbonization and physical or chemical activation. During the carbonization stage, the precursor material is converted into activated carbon through a thermal decomposition process, which results in a low adsorption efficiency of the produced material Yang & Qiu 2010). On the other hand, activation increases the efficiency and improves the pore structure. Activation can be achieved through traditional heating methods or microwave-assisted techniques (Liew et al. 2018). The advantages of the microwave-assisted method, compared to the traditional approach, include shorter processing times and the more homogeneous conversion of biomass into activated carbon (Hesas et al. 2013). Microwave heating increases the carbon yield, improves the quality of the activated carbon, ensures high energy efficiency, and makes the technique environmentally friendly by minimizing the formation of hazardous substances and emissions (Xie et al. 2014). Peanut shells are an abundant agricultural waste. According to the report of the Food and Agriculture Organization of the United Nations, global peanut production is 46 million tons. Shells account for 20% of the weight of peanuts, or 9.2 million tons. Peanut shells contain 37.1% cellulose, 33.4% hemicellulose and 15% lignin. To produce biofuels from lignocellulosic materials such as peanut shells, physical, chemical, physico-chemical and biological pretreatments are used to break down the complex structure of the biomass, releasing cellulose and hemicellulose (Bostancı et al. 2019).

In this study, microwave-assisted activated carbon (MW-AC) was prepared from peanut shells by the chemical activation method and used for the first time in the adsorption of benzene vapor. In the microwave-assisted activated carbon preparation reported in the literature, microwave treatment is usually applied in the activation stage. However, in this study, unlike in the literature, the microwave treatment was applied in the impregnation stage of the activated carbon production process, which highlights the innovative aspect of our work. The main purpose of using the microwave treatment in the impregnation stage is to both reduce the impregnation time and to allow more effective penetration of the activating agent into the internal parts of the raw material.

## Experimental section

### Chemicals

Zinc chloride (ZnCl_2_), hydrochloric acid (HCl), sodium thiosulfate pentahydrate (Na_2_S_2_O_3_.5H_2_O), iodine (I_2_), benzene (C_6_H_6_) and, potassium iodine (KI) utilized in the experimental investigations were provided by Merck and were of analytical purity. Distilled water was used as the experimental solvent. Peanut shells were sourced from the market in Osmaniye, Turkey.

### Microwave-assisted fabrication of activated carbon

**Fig. 1:**
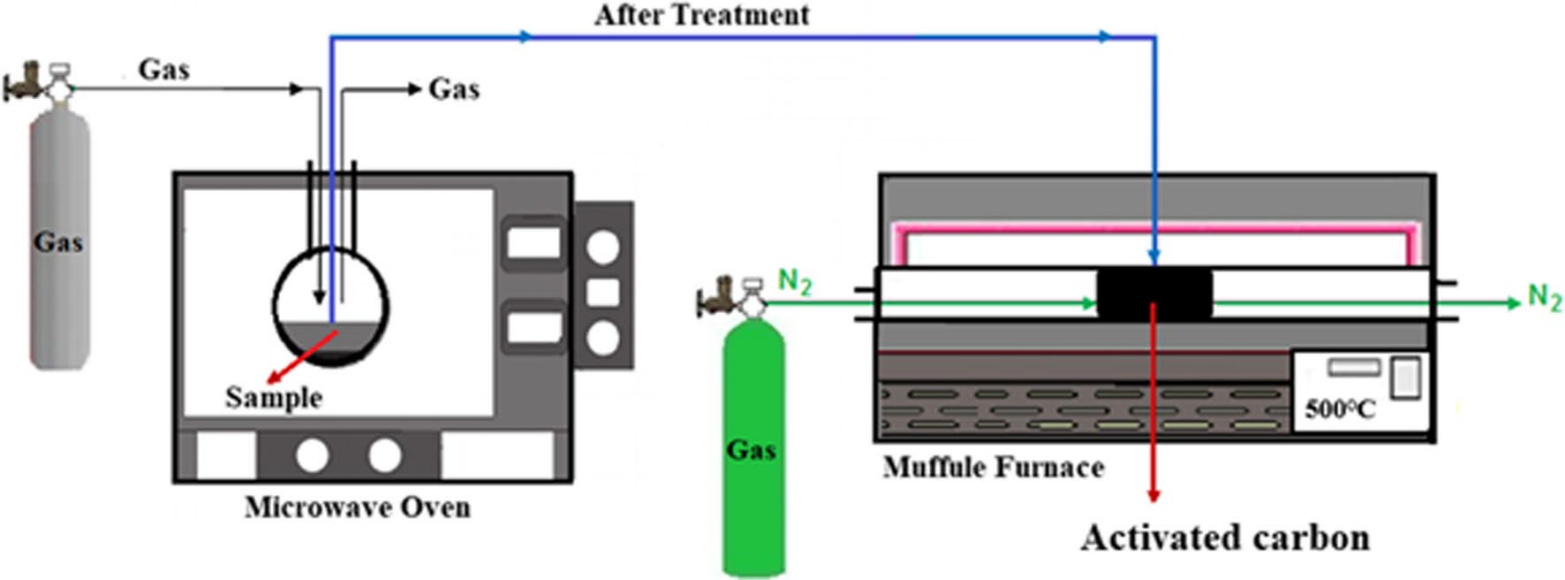
Schematic diagram of the experimental setup for MW-AC fabrication

The fabrication of MW-AC followed the experimental setup outlined in Fig. 1 (Batur et al. 2023; Yildiz et al. 2024). The process consisted of two steps: microwave treatment and muffle furnace heating. First, peanut shells were ground to a particle size of -500 + 350 μm, washed with distilled water, and dried at 80 °C. In the MW-AC chemical activation method, 3 g of ground peanut shells were combined with 3 g of ZnCl_2_ activator dissolved in 2 mL of distilled water. The impregnation of the activator into the peanut shells was conducted in a microwave environment. During the chemical activation process in the muffle furnace, the impregnated samples were heated at an activation temperature of 500 °C for 45 min in a nitrogen (N_2_) atmosphere, resulting in the fabrication of MW-AC. The MW-AC obtained was washed with 0.5 M HCl followed by rinsing with hot distilled water until pH 6–7 was reached. During the MW-AC fabrication process, the effects of impregnation rate, different gases in the microwave, microwave power and microwave duration were evaluated. The MW-AC obtained under optimum conditions was then used for dynamic adsorption of benzene vapor.

### Characterizations

The iodine number, a fundamental parameter used to assess the performance of the MW-AC produced, is critical to the characterization of the activated carbon (AC), particularly in determining the surface area of pores larger than 1 nm in size. The procedure for determining the iodine number follows the guidelines of the American Society for Testing and Materials (ASTM 2006). The iodine number (mg/g) was calculated using Eq. (1) (ASTM 2006):

1 $$Iodine number=\frac{({V}_{2}-{V}_{1})\times 127\times N\times 40}{w\times {V}_{2}}$$

Here, V_1_ (mL) = Na_2_S_2_O_3_.5H_2_O amount used in titration after iodine adsorption of MW-AC, V_2_ (mL) = Na_2_S_2_O_3_.5H_2_O amount used in titration for 0.1 N iodine solution, N (N) = iodine solution concentration, w (g) = MW-AC amount.

The structural and morphological properties of the MW-AC with the highest iodine number were evaluated by scanning electron microscope (SEM, JEOL/JSM-6610 instrument), Fourier transform infrared (FTIR, Perkin Elmer 100 FTIR spectrometer), and Brunauer–Emmett–Teller (BET, Quantachrome Nova 1200 series instrument) analysis. The FTIR analysis was performed in the wavenumber range of 4000–500 cm^−1^. BET analysis, pore size, and pore volume were determined by N_2_ adsorption/desorption at 77 K.

### Adsorption experiments

Dynamic adsorption studies were conducted using the system outlined in our previously published work (Kutluay et al. 2019a). Adsorption experiments were performed in a Pyrex glass fixed bed reactor (adsorber) at atmospheric pressure, with an internal diameter of 0.9 cm and a height of 16 cm. During the experiment, benzene vapor generated by a PID-controlled heated thermostat was passed over the adsorbent (m = 0.05 g) in the adsorber with a flow of N_2_ (F = 100 mL/min). Gas chromatography (GC) with a flame ionization detector (FID) was employed to measure both the inlet and outlet concentrations. The adsorption capacity was calculated using Eq. (2) (Zhao et al. 2015):

2 $$q=\frac{F}{m}\left({C}_{inlet}t-\underset{0}{\overset{t}{\int }}{C}_{outlet}dt\right)$$

Here, q (mg/g) = adsorption capacity of benzene, F (L/min) = flow rate of N_2_, m (g) = amount of MW-AC adsorbent, C_inlet_ (ppm) = inlet concentration of benzene vapor, C_outlet_ (ppm) = outlet concentration of benzene vapor, t (min) = contact duration.

### Reusability test

The reusability of the MW-AC adsorbent over five consecutive adsorption–desorption cycles was assessed. In the desorption experiment, N_2_ was circulated over the adsorbent saturated with benzene vapor for a contact duration of 90 min at the boiling temperature of benzene, which was set at 80 °C. To desorb the benzene vapor adsorbed on the adsorbent, after the adsorption process, the sample was positioned in an oven heated to the boiling point of benzene and N_2_ was passed over the sample, thus carrying out the desorption process. The reuse efficiency (RE, %) was computed using Eq. (3) (Rajabi et al. 2021):

3 $$RE (\%)=\frac{{q}_{e}(n)}{{q}_{i}}\times 100$$

Here, q_i_ (mg/g) = initial adsorption capacity before the first cycle (from Eq. 2), q_e_(n) (mg/g) = adsorption capacity at the nth cycle (from Eq. 2).

### Experimental design for benzene vapor adsorption

The Box-Behnken approach within the RSM was employed in the design of experiments for the adsorption of benzene vapor using a MW-AC adsorbent. The design aimed to maximize the adsorption capacity of benzene by considering process parameters such as contact duration, inlet concentration, and temperature. The values corresponding to the design points of the studied independent variables are summarized in Table 1. The relationship between the adsorption capacity (q) response and the independent variables of contact duration (x_1_), inlet concentration (x_2_) and temperature (x_3_) was modeled using Eq. (4) (Fouladian & Behbahani 2015):

**Table 1:** Three-variable experimental design levels

Independent variables-code	Levels in the Box-Behnken design		
	-1	0	+ 1
Contact duration: x_1_ (min)	40	60	80
Inlet concentration: x_2_ (ppm)	10	15	20
Temperature: x_3_ (°C)	25	32.5	40

4 $$Y={\beta }_{0}+{\sum }_{i=1}^{n}{\beta }_{i}{x}_{i}+{\sum }_{i=1}^{n}{\beta }_{ii}{x}_{i}^{2}+{\sum }_{i=1}^{n}{{\sum }_{j=1}^{n}{\beta }_{ij}x}_{i}{x}_{j}$$

Here, Y (mg/g) = adsorption capacity, x_i_ (i = 1, 2, and 3; j = 1, 2, 3) = independent variables coded values, β_0_ and β_i_ (i = 1, 2, 3; j = 1, 2, 3) = predicted quadratic model coefficients.

### Statistical analysis

The mean relative absolute error (MRAE) and normalized root mean square error (NRMSE) error metrics were used to determine the best fitting models by the deviation between experimental and modelled values. The MRAE model, represented by Eq. (5), was utilized for non-zero data points, while the NRMSE model, defined by Eq. (6), was employed to assess the overall error in predicting the experimental values (Tefera et al. 2014).

5 $$\mathrm{MRAE model}=\frac{1}{N}\sum_{1}^{N}\left(\frac{\left|\mathrm{experimental value }-\mathrm{ modelled value}\right|}{\mathrm{experimental value}}\times 100\right)$$

6 $$\mathrm{NRMSE model}=\frac{\sqrt{\frac{1}{{\text{N}}}\sum_{1}^{{\text{N}}}{ \left(\mathrm{experimental value }-\mathrm{ modelled value}\right)}^{2}}}{\mathrm{experimental value}}\times 100$$

## Results and discussion

### Fabrication of MW-AC

**Fig. 2:**
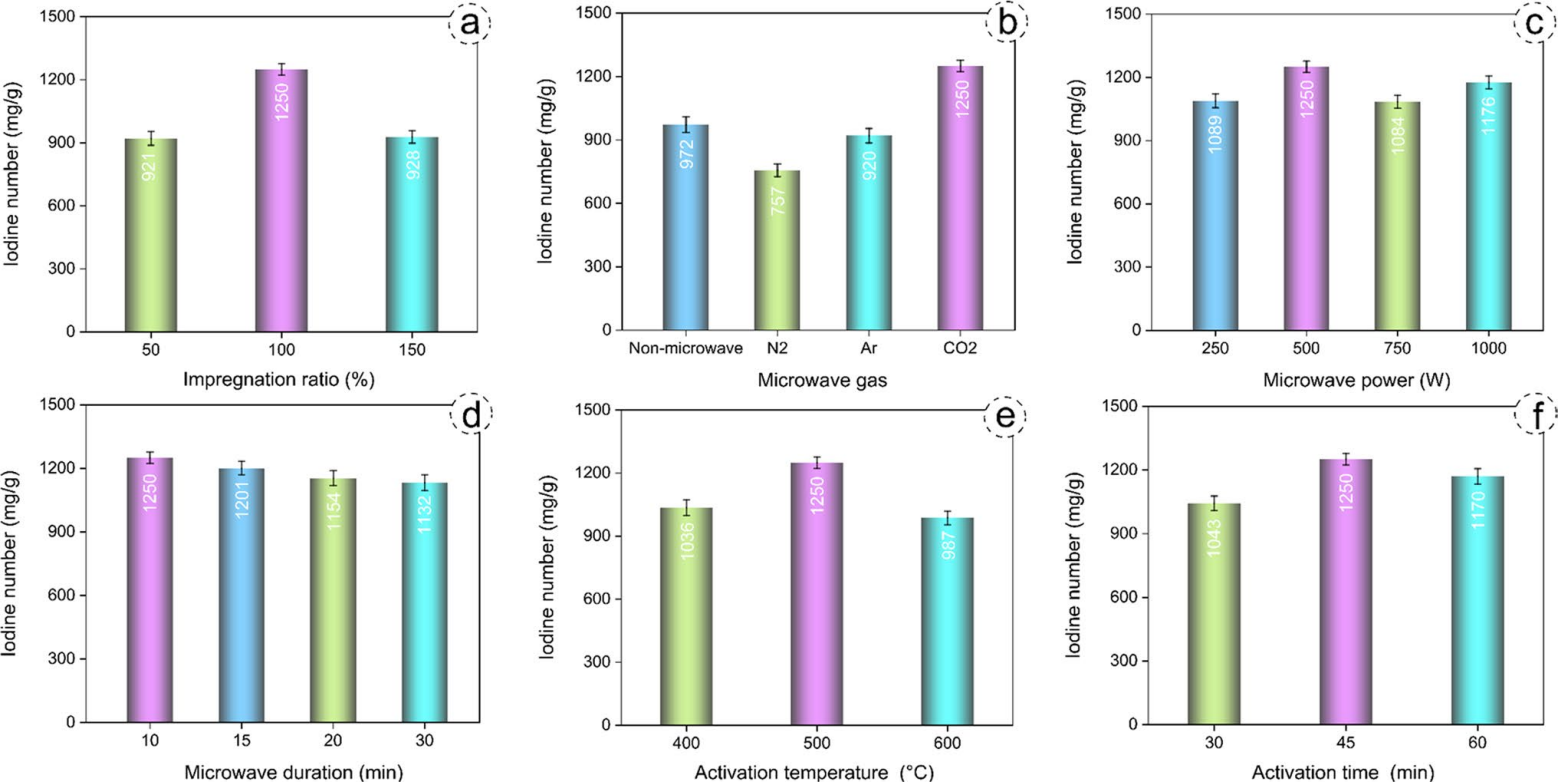
The influence of impregnation ratio (**a**), gases in the microwave environment (**b**), microwave power (**c**), microwave duration (**d**), activation temperature (**e**), and activation time (**f**) on the iodine number of MW-AC

In the preparation of activated carbon, the impregnation ratio stands out as a critical parameter that influences the efficiency, surface area, total pore volume and pore size. These properties play a key role in determining the effectiveness of activated carbon in various industrial applications (Misran et al. 2020). To investigate the influence of the impregnation ratio, experiments were conducted under specific conditions: 500 W microwave power, 10 min microwave duration, CO_2_ as the gas in the microwave environment, 500 °C activation temperature, and 45 min activation time. The results, depicting the correlation between the impregnation ratio and the variation of the iodine number, are illustrated in Fig. 2a. As can be seen from Fig. 2a, it is evident that as the impregnation ratio increases from 50 to 100%, the iodine number also increases, whereas at 150% impregnation ratio, the iodine number decreases. The probable reason for this phenomenon could be that at low activator levels, full interaction with the raw material may not occur, resulting in incomplete pore formation. On the other hand, with a high activator amount, it is assumed that the activated carbon may cause the pores to form in a macro structure.

After determining the optimum impregnation ratio, the influence of Ar, N_2_ and CO_2_ gases in the microwave environment was investigated under the conditions of a 100% impregnation ratio, 500 W microwave power, 10 min microwave duration, 500 °C activation temperature, and 45 min activation time. The results, depicting the correlation between the gases in the microwave environment and the variation of the iodine number, are illustrated in Fig. 2b. The possible reason for a higher iodine number, as depicted in Fig. 2b, in activated carbon obtained in the presence of CO_2_ gas is considered to be the weakening of the bonds between the activator and the raw material in the microwave environment of CO_2_. This finding is consistent with a study where the microwave effect of CO_2_ atmosphere was found to be more effective in the production of activated carbon from almonds (Teğin et al. 2020). CO_2_ as the gas in the microwave environment was used to study the influence of other parameters. The observed low iodine number in the presence of Ar and N_2_ gases can be attributed to their interference with the penetration of microwave power into the internal parts of the raw material. In essence, these gases reduced the effect of the microwave treatment (Şahin et al. 2013).

The influence of microwave power was investigated under the conditions of a 10 min microwave duration, CO_2_ as the gas in the microwave environment, 500 °C activation temperature, and 45 min activation time. The results, depicting the correlation between the microwave power and the variation of the iodine number, are illustrated in Fig. 2c. As the microwave power increases from 250 to 500 W, the iodine number increases; however, when the microwave power is greater than 500 W, the iodine number decreases. It is assumed that the microwave power of 250 W is not sufficient to break the bonds between the raw material and the activator, and that the microwave power of 500 W completely weakens the bonds between the activator and the raw material. If the microwave power is greater than 500 W, it is assumed to cause the pores in the structure of the raw material to collapse.

The influence of microwave duration was investigated under the conditions of 500 W microwave power, CO_2_ as the gas in the microwave environment, 500 °C activation temperature, and 45 min activation time. The results, depicting the correlation between the microwave duration and the variation of the iodine number, are illustrated in Fig. 2d. At a microwave duration of 10 min, the iodine number was determined to be 1250 mg/g, whereas at a microwave duration of 20 min, this value was found to be 1154 mg/g. With a microwave duration of 30 min, the iodine number was determined to be 1132 mg/g. The possible reason for this phenomenon is that the activator disrupts the potential pore structure when the microwave duration exceeds 15 min. In other words, prolonged exposure of the raw material to the microwave environment may cause an excessive reduction in the viscosity of the activator, resulting in a deterioration in the pore structure of the raw material.

After determining the microwave environment parameters that give the highest iodine number, the activation parameters were investigated. The influence of the activation temperature was studied under the conditions of 500 W microwave power, 10 min microwave duration, CO_2_ as the gas in the microwave environment, and 45 min activation time. The results, depicting the correlation between the activation temperature and the variation of the iodine number, are illustrated in Fig. 2e. As can be seen in Fig. 2e, an increase in activation temperature from 400 °C to 500 °C resulted in an initial increase in iodine number followed by a subsequent decrease. The possible reason for this phenomenon is that the activation temperature of 400 °C is not sufficient to open the pores in the MW-AC structure, and an activation temperature of 600 °C may cause the transformation of micropores into macropores.

The influence of the activation time was investigated under the conditions of 500 W microwave power, 10 min microwave duration, CO_2_ as the gas in the microwave environment, and 500 °C activation temperature. The results, depicting the correlation between the activation time and the variation of the iodine number, are illustrated in Fig. 2f. When the activation times were 30, 45, and 60 min, the iodine numbers were determined to be 1043, 1250, and 987 mg/g, respectively. The possible reason for this is that an activation time of 30 min may not be sufficient to completely open the pores in the MW-AC structure. When the activation time is increased to 60 min, it is assumed that the micropores of the MW-AC transform into mesopores, leading to the collapse of the MW-AC pore structure.

### Characterization of MW-AC

The structural and morphological properties of the optimized MW-AC (impregnation ratio = 100%, CO_2_ as the gas in the microwave environment, microwave power = 500 W, microwave duration = 10 min, activation temperature = 500 °C, and activation time = 45 min) were assessed through SEM, FTIR, and BET analysis.

**Fig. 3:**
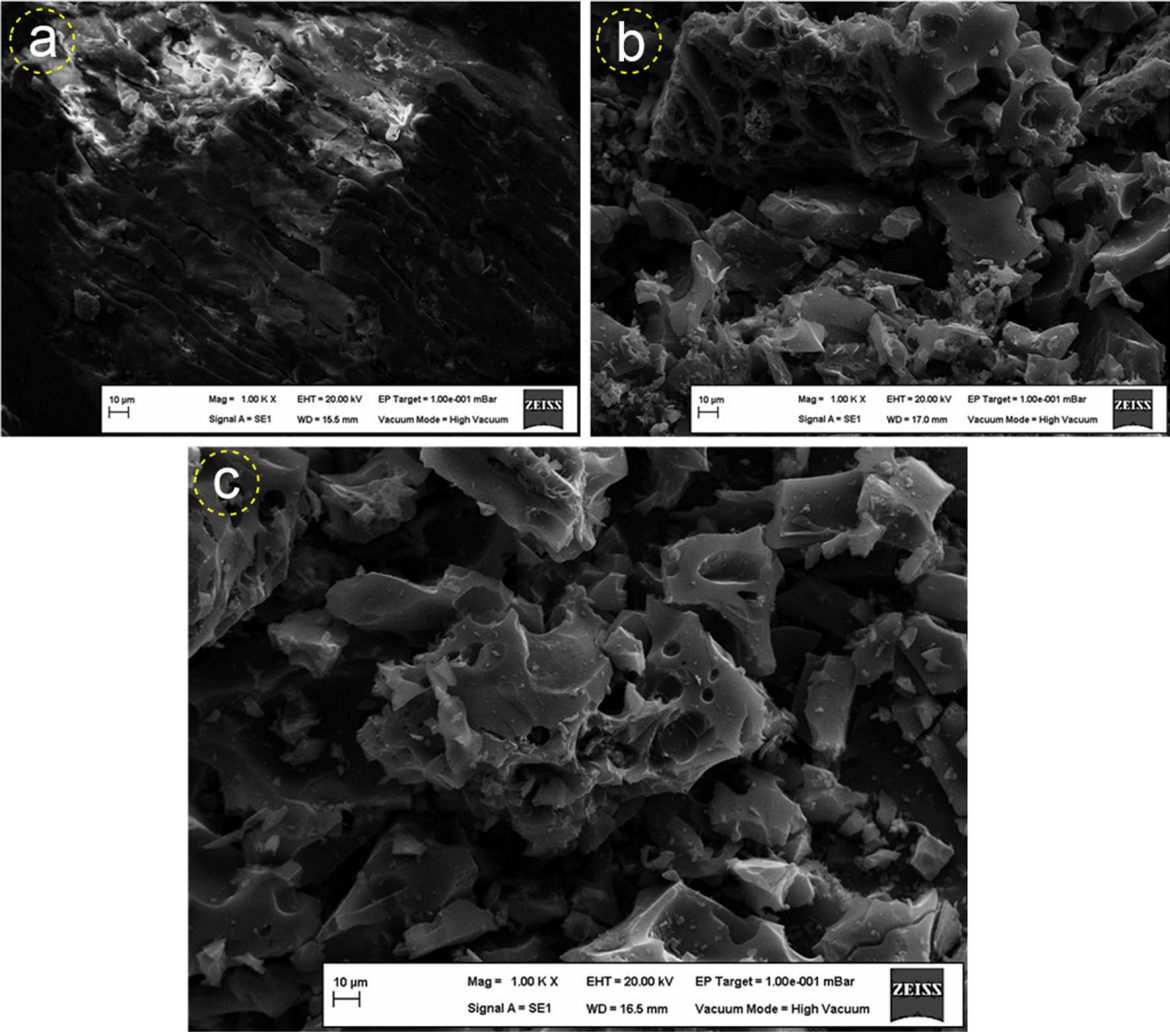
SEM images of peanut shell (**a**), AC (**b**) and MWAC (**c**)

Figure 3 presents SEM images of peanut shell, activated carbon (AC) produced without microwave assistance, and microwave-assisted activated carbon (MW-AC). As shown in Fig. 3a, the surface of the peanut shell is generally characterized by low porosity and roughness. In contrast, the surface of MW-AC is porous and smoother, indicating that the surface of MW-AC is more porous and smoother compared to the peanut shell (Fig. 3c). This observation suggests that microwave energy enhances the microporous structure of AC. The microporous structures of MW-AC are more pronounced compared to AC, indicating that MW-AC is rich in micropores (Fig. 3b, c). On the other hand, the literature reports that the surface of AC prepared using sawdust with ZnCl_2_ activator undergoes microstructure formation at 500 °C, and pores collapse at higher temperatures (Lin et al. 2021).

**Fig. 4:**
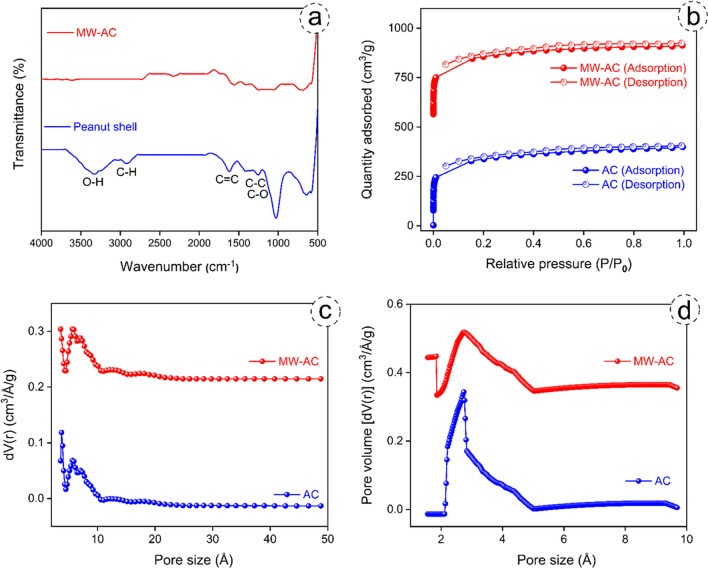
FTIR spectra of peanut shell and MW-AC (**a**), and N_2_ adsorption and desorption hysteresis (**b**), DFT pore size distributions (**c**), HK differential pore volume plots (**d**) for AC and MW-AC

The FTIR results of peanut shell and MW-AC are shown in Fig. 4a. From Fig. 4a, it can be seen that the peanut shell structure contains several functional groups. The peak at 3400 cm^-1^ indicates the presence of OH^−^ functional groups bonded by hydrogen bonds (Ece et al. 2021a; Ekinci et al. 2023; Kutluay et al. 2022). The peak at 2900 cm^-1^ is attributed to the C-H functional group derived from methyl groups, and the peak at 1600 cm^-1^ indicates the presence of C $=$ C bonds derived from olefinic groups. The peak at 1266 cm^-1^ indicates the presence of C–C and C-O functional groups. Peaks below 1000 cm^-1^ indicate the presence of functional groups derived from the aromatic ring (Wibawa et al. 2020). From Fig. 4a, it is clear that many of the functional groups present in the peanut shell structure are not observed in the MW-AC structure. The possible reason for this is believed to be the weakening of the bonds between the activator and the raw material due to the microwave power (Teğin et al. 2020).

The N_2_ adsorption/desorption method was employed to analyze the pore size distribution of both AC and MW-AC. In Fig. 4b-d, the BET measurement results depict the N_2_ adsorption and desorption isotherms, which facilitate the evaluation of surface parameters such as surface area, pore volume, and pore size for AC and MW-AC. Figure 4b illustrates that both AC and MW-AC exhibit H3 hysteresis cycle type I curves (Ece & Kutluay 2022) according to the International Union of Pure and Applied Chemistry (IUPAC) classification. The Density Functional Theory (DFT) method was utilized to calculate the size distributions of AC and MW-AC, revealing pore sizes of 0.75 nm and 1.13 nm, respectively (Fig. 4c). In addition, Fig. 4d shows the micropore distributions estimated using the Horvath-Kawazoe (HK) method. The micropore volumes for AC and MW-AC were determined to be 0.507 cm^3^/g and 0.522 cm^3^/g, respectively, by t-plot calculation. Considering the total pore volumes of AC (0.617 cm^3^/g) and MW-AC (0.631 cm^3^/g), the ratios of micropores to total porosity were found to be 82.17% and 82.73%, respectively. Furthermore, the BET specific surface area was calculated to be 1119.42 m^2^/g for AC and 1204.90 m^2^/g for MW-AC. These results are in agreement with previously reported literature, confirming the consistency of the results (Kim et al. 2020). Table 2 provides a summary of the BET specific surface area, pore volume, and pore size for both AC and MW-AC.

**Table 2:** Textural properties of AC and MW-AC

Sample	Surface area (m^2^/g) ^**a**^	Total pore volume (cm^3^/g) ^**a**^	Micropore volume (cm^3^/g) ^**a**^	Mesopore volume (cm^3^/g) ^**a**^	Pore size (nm) ^**a**^
AC	1119.42	0.617	0.507	0.110	0.75
MW-AC	1204.90	0.631	0.522	0.109	1.13

^**a**^ Specific surface area calculated by BET method; pore size and total pore volume calculated by DFT method; micropore volume calculated by HK method

### Model validation, impact of operating factors and optimization studies for benzene adsorption

**Fig. 5:**
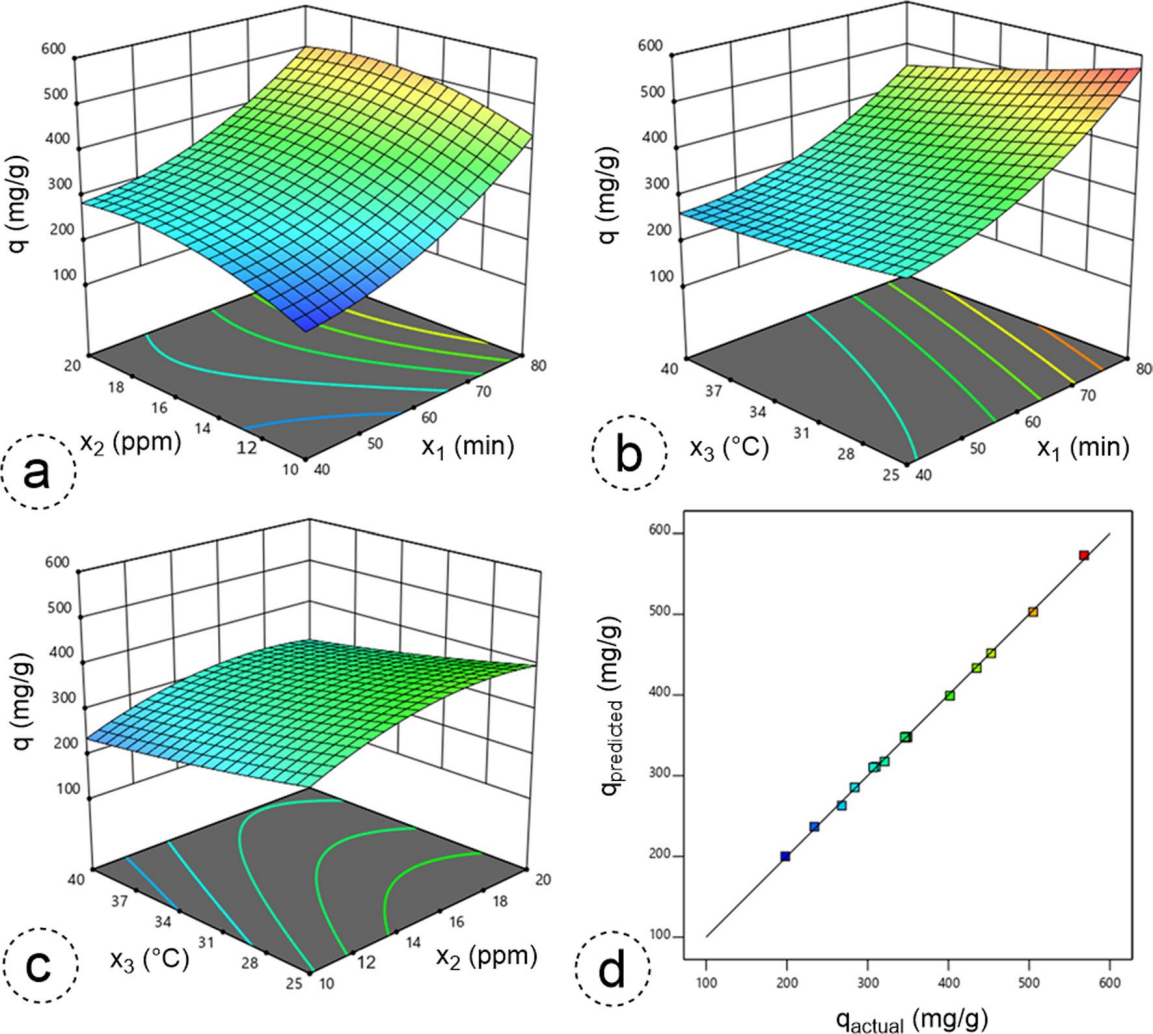
3D response surface plots illustrating the combined impacts of dual independent variables: (**a**) contact duration versus inlet concentration (x_1_—x_2_), (**b**) contact duration versus temperature (x_1_—x_3_) and (**c**) inlet concentration versus temperature (x_2_—x_3_), and (**d**) the plots comparing the actual and predicted adsorption capacity (q) for the adsorption of benzene

At this stage, we utilized the Box-Behnken experimental design approach within RSM to experimentally design and optimize operational factors—such as contact duration, inlet concentration and temperature—that impact the benzene adsorption process. Analysis of variance (ANOVA) was conducted on benzene adsorption using a proposed second-degree quadratic regression model. This analysis evaluated the agreement between the experimental data and the predicted model by assessing the adequacy of various parameters. The calculated critical F-value was 983.69, indicating a substantial representation and validity of the model with respect to the experimental results (Baytar et al. 2020). In addition, the probability of obtaining such a significant F-value by chance was only 0.01%, reinforcing the reliability of the model. P-values were used to indicate the significance of model terms. Values below 0.0500 indicate the importance of model terms, while values above 0.1000 indicate insignificance (Kutluay 2021). If a large number of insignificant model terms are present (excluding those essential to the hierarchy), the model may be trimmed or revised. The ANOVA results showed that contact time had the highest F-value (6255.62) with a low P-value (< 0.0001), confirming its prominence as the most influential factor for benzene adsorption (Temel & Kutluay 2020). Ideally, in a developed model, the difference between predicted R^2^ and adjusted R^2^ values should be less than 0.2. The obtained predicted R^2^ value of 0.988 and adjusted R^2^ value of 0.998 for benzene adsorption validate the suitability of the model with experimental data. Furthermore, to assess the reliability of the model, a comparison was made between the actual and predicted values of the benzene adsorption capacity (q) (Fig. 5d). The results showed a close agreement between these values, confirming the accuracy of the proposed model for benzene adsorption (Temel and Kutluay 2020).

Figure 5 illustrates the impacts of operational variables, namely contact duration (x_1_), inlet concentration (x_2_) and temperature (x_3_), on the benzene adsorption capacity (q). Observing Fig. 5a, it is clear that, at constant temperature, an increase in either contact duration or inlet concentration independently leads to an increase in adsorption capacity. The ANOVA analysis carried out specifically highlights the contact duration as a more important determinant in the process. Furthermore, it was found that the inlet concentration increases the adsorption capacity up to about 18 ppm, beyond which no significant changes occur, suggesting saturation of the active sites on the adsorbent surface by benzene vapor. In Fig. 5b, the trend shows an increase in benzene adsorption capacity with prolonged contact duration, but a decrease with increasing temperature. Given the exothermic nature of gas adsorption, the negative correlation between temperature and adsorption is expected. The ANOVA results confirm the significant effect of temperature on the process, similar to that of contact duration. Figure 5c shows a decrease in adsorption capacity with increasing temperature, while no significant variation in inlet concentration is observed. These results emphasise the greater impact of temperature compared to inlet concentration, which is consistent with the ANOVA results (Baytar et al. 2020; Ece et al. 2020).

The main objective of this study was to optimize the adsorption process in order to achieve the highest possible benzene adsorption capacity by using various experimental settings. During the adsorption process, the Box-Behnken design method was effectively applied to determine the peak adsorption capacity of the MW-AC adsorbent for benzene and to identify numerical values for the most efficient factors. The established optimum conditions resulted in a maximum adsorption capacity of 568.34 mg/g for benzene, according to the recommended model, with a contact duration of 79.67 min, an inlet concentration of 17.50 ppm and a temperature of 26.02 °C. In addition, a validation experiment carried out under these ideal conditions confirmed a maximum adsorption capacity of 557.59 mg/g for benzene. These results show considerable consistency, indicating strong agreement between the proposed model and the experimentally derived results.

### Adsorption kinetic and isotherm models

The adsorption kinetic was elucidated by fitting the experimental data to the pseudo-first-order model represented by Eq. (7) (Kutluay et al. 2019b), the pseudo-second-order model represented by Eq. (8) (Temel and Kutluay 2020) and the intraparticle diffusion model represented by Eq. (9) (Batur and Kutluay 2022):

7 $${q}_{t}={q}_{e}(1-{e}^{-{k}_{1} t})$$

8 $${q}_{t}= \frac{{k}_{2}{{ q}_{e}}^{2} t}{1+{k}_{2 }{q}_{e} t}$$

9 $${q}_{t}={k}_{\text{id }}{t}^{0.5}+C$$

Here, q_e_ (mg/g) and q_t_ (mg/g) are the respective adsorption capacities observed at equilibrium and at a contact duration t (min). k_1_ (1/min), k_2_ (g/mg/min) and k_id_ (mg/g/min^0.5^) are the adsorption rate constants corresponding to the pseudo-first-order, pseudo-second-order and intraparticle diffusion models respectively. In addition, C (mg/g) represents a constant associated with the boundary layer thickness.

**Fig. 6:**
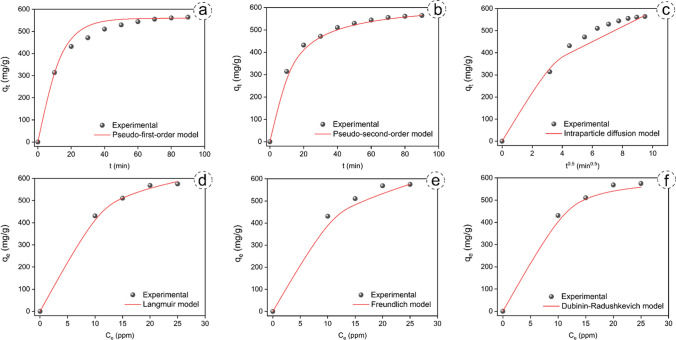
Fitting plots of kinetic models including pseudo-first-order (**a**), pseudo-second-order (**b**) and intraparticle diffusion (**c**), and isotherm models including Langmuir (d), Freundlich (**e**) and Dubinin-Radushkevich (**f**) for benzene adsorption

The kinetic model parameters were obtained from experiments conducted at an adsorption temperature of 26 °C, using an inlet concentration of 20 ppm and varying contact durations (10–90 min). Plots illustrating the kinetic models are shown in Fig. 6a-c and detailed parameters calculated by least squares regression are listed in Table 3. Analysis revealed high regression coefficient (R^2^ = 0.993) and low values of MRAE (9.34) and NRMSE (9.16) for the pseudo-second-order kinetic model compared to the others (Table 3). The pseudo-second-order kinetic model suggests that the adsorption process is a chemical process. An intraparticle diffusion model was used to investigate the role of diffusion in adsorption. Its application to the data showed that when plotting q_t_ versus t^0.5^, a linear curve passing through the origin indicates sole control by intraparticle diffusion. However, as depicted in Fig. 6c, the curve for the intraparticle diffusion kinetic model isn't linear or passes through the origin. This behavior suggests the involvement of multi-step mechanisms in the adsorption process (Li et al. 2018).

**Table 3:** Parameters of kinetic models including pseudo-first-order, pseudo-second-order and intraparticle diffusion, and isotherm models including Langmuir, Freundlich and Dubinin-Radushkevich for benzene adsorption

Kinetic models / Parameters																				
Pseudo-first-order model							Pseudo-second-order model						Intraparticle diffusion model							
q_e_ (mg/g)		k_1_ (1/min)	R^2^	MRAE		NRMSE	q_e_ (mg/g)		k_2_ (g/min/mg)	R^2^	MRAE	NRMSE	k_id_ (mg/g/min^0.5^)		C	R^2^		MRAE	NRMSE	
559.62		0.097	0.832	50.45		45.07	626.87		1.6 $\times$ 10^–4^	0.993	9.34	9.16	34.091		245.83	0.809		59.54	47.24	
Isotherm models / Parameters																				
Langmuir model							Freundlich model						Dubinin-Radushkevich model							
q_max_ (mg/g)	K_L_ (L/mg)		R_L_	R^2^	MRAE	NRMSE	K_F_ (mg/g) (L/mg)^1/n^	n		R^2^	MRAE	NRMSE	q_m_ (mg/g)	K (mol^2^/J^2^)		E (kJ/mol)	R^2^	MRAE		NRMSE
738.53	0.154		0.2–0.4	0.982	8.66	10.57	194.21	2.96		0.938	13.18	18.79	587.56	5.37 $\times$ 10^–6^		0.32	0.970	8.71		12.90

The adsorption equilibrium mechanism was described by fitting the experimental data to the Langmuir isotherm model represented by Eq. (10) (Hazzaa & Hussein 2015), the Freundlich isotherm model represented by Eq. (11) (Erol et al. 2019) and the Dubinin-Radushkevich isotherm model represented by Eqs. (11–14) (Ganguly et al. 2020):

10 $${q}_{e}=\frac{{{q}_{max} K}_{L} {C}_{e}}{{1+ K}_{L} {C}_{e}}$$

11 $${q}_{e}={K}_{f} {{C}_{e}}^\frac{1}{n}$$

12 $${q}_{e}={q}_{m}\mathit{exp}\left(-K{\varepsilon }^{2}\right)$$

13 $$\varepsilon =RT {\text{ln}}\left(1+\frac{1}{{C}_{e}}\right)$$

14 $$E=\frac{1}{\sqrt{2K}}$$

Here, C_e_ (ppm) is the equilibrium concentration, q_e_ (mg/g) is the adsorption capacity at this equilibrium state. q_max_ (mg/g) and K_L_ (L/mg) are the maximum adsorption capacity and the Langmuir constant, respectively. K_F_ ((mg/g)(L/g)^1/n^) and n describe parameters related to the adsorption capacity and intensity, where 1/n is the factor representing the heterogeneity. q_m_ (mg/g) represents the Dubinin-Radushkevich adsorption capacity, K (mol^2^/J^2^) is a constant related to the adsorption energy and ε (J/mol) refers to the Polanyi potential.

Experimental data were collected at an adsorption temperature of 26 °C, over a contact duration of 90 min, with varying inlet concentrations (10–25 ppm). Plots representing the isotherm models are in Fig. 6d-f and detailed parameters calculated by least squares regression are listed in Table 3. The Langmuir model showed a high R^2^ (0.982) and low values of MRAE (8.66) and NRMSE (10.57) compared to other models (Table 3). The results indicate that benzene adsorption is better described by the Langmuir model (Fig. 6d-f). This suggests that the adsorption process is a chemical process. The calculated n-value for the Freundlich model was 2.96, indicating a physical adsorption process (Zou et al. 2019). Furthermore, the calculated E-value (0.32 kJ/mol) of < 8 kJ/mol for the Dubinin-Radushkevich model indicates a physical interaction mechanism during adsorption (Temel & Kutluay 2020) Fig. 6.

### Regeneration and reusability of MW-AC

**Fig. 7:**
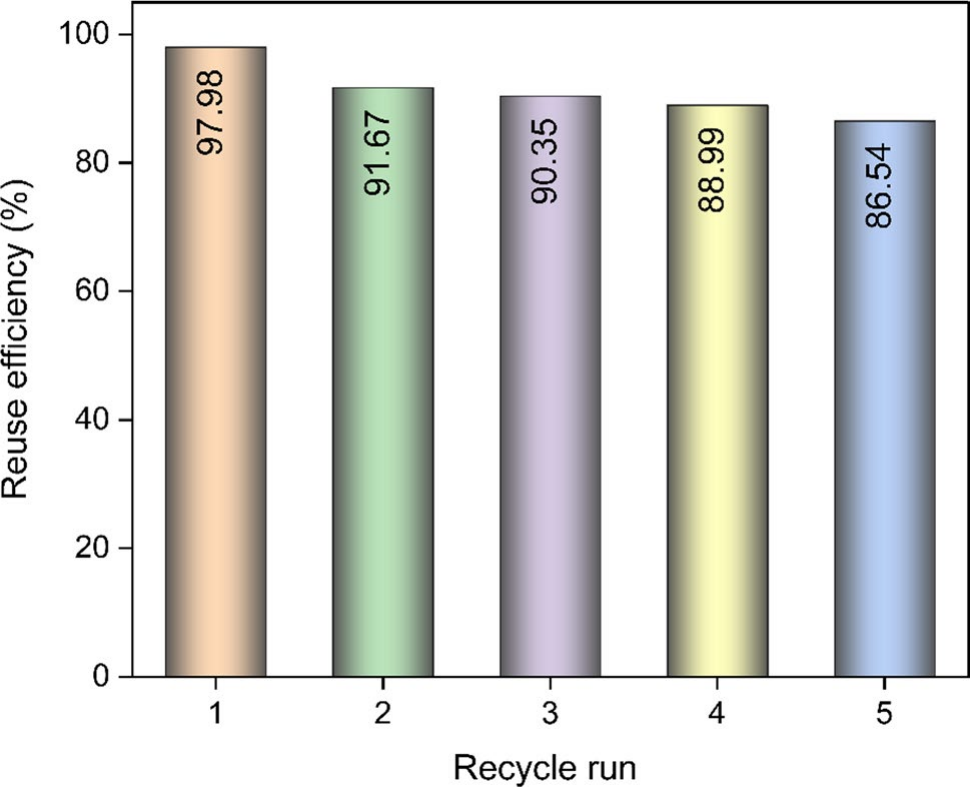
Reusability and adsorption stability of MW-AC for benzene vapor adsorption

The reusability and adsorption stability of the adsorbent play a crucial role in assessing the practical applicability of the developed method. Indeed, the reusability performance of the adsorbent is a paramount criterion for practical applications and influences the overall cost of the adsorption process. The reuse efficiency of MW-AC for benzene vapor adsorption was determined after five consecutive recycling cycles. As shown in Fig. 7, MW-AC exhibited a reuse efficiency of 86.54% for benzene vapor after undergoing five consecutive recycling processes. The strong reusability and adsorption stability suggest the presence of van der Waals forces or π-π interactions between the MW-AC adsorbent and the benzene adsorbate (Gan et al. 2021, Kutluay & Temel 2021). On the contrary, incomplete regeneration processes can be attributed to the formation of permanent bonds on the adsorbed surface, resulting in an irreversible conversion of adsorbed groups. In other words, some chemically bound adsorbates that are not desorbed during regeneration may block the pores, leading to residue formation (Auta & Hameed 2014).

### Comparison of MW-AC adsorption capacity with literature

Table 4 provides a brief comparison between the results of this study and previous research. The evaluation of the adsorption capacities presented in Table 4 highlights the remarkable potential of MW-AC in comparison to other adsorbents documented in the literature. The superior adsorption capacity of the MW-AC adsorbent underlines its promising efficacy for the removal of VOCs in industrial processes.

**Table 4:** Comparison of MW-AC adsorption capacity with literature for benzene vapor

Adsorbent	Adsorption capacity (mg/g)	Reference
Biochar	54.60	(Kumar et al. 2020)
Cu-3@MIL-101(Cr)	114.40	(Wang et al. 2018)
NHPC	151.00	(Tang et al. 2020)
AC	161.42	(Wang et al. 2004)
Fe_3_O_4_@SiO_2_	197.50	(Ece et al. 2021b)
rGO	276.40	(Yu et al. 2018)
MFOF-1a	356.18	(Zhang et al. 2016)
PDMS/AC-250	360.00	(Liu et al. 2016)
AC	437.36	(Baytar et al. 2020)
DBCB-AC	503.18	(Batur & Kutluay 2022)
AC	494.53	This study
MW-AC	568.34	This study

## Conclusions

In the present study, MW-AC was successfully fabricated from peanut shells uzing a ZnCl_2_ activator, and demonstrated its effectiveness in eliminating benzene vapor, a VOC. The fabrication process involved a comprehensive investigation of various factors, including impregnation ratio, gases in the microwave environment, microwave power, microwave duration, activation temperature, and activation time. The optimum conditions yielded a remarkable iodine number of 1250 mg/g, which was achieved with a 100% impregnation ratio, CO_2_ as gas in the microwave environment, 500 W microwave power, 10 min microwave duration, 500 °C activation temperature, and 45 min activation time. The structural and morphological properties of the optimized MW-AC were thoroughly evaluated by SEM, FTIR, and BET analysis. The significant BET surface area of 1204.90 m^2^/g highlighted the effectiveness of the developed MW-AC as an adsorbent. The dynamic adsorption process of benzene on the optimized MW-AC, which was modeled using the Box-Behnken approach within the RSM, revealed a maximum adsorption capacity of 568.34 mg/g at a contact duration of 79.67 min, an inlet concentration of 17.50 ppm, and a temperature of 26.02 °C. Combined with the results of pseudo-second-order kinetic model and Langmuir, Freundlich and Dubinin-Radushkevich isotherm models, both chemical adsorption and physical adsorption are exist in the adsorption process of benzene vapor adsorbed by MW-AC. After five consecutive recycling processes, MW-AC showed a recycling efficiency of 86.54% for benzene vapor. These results underline the promising potential of MW-AC as a candidate for VOC treatment in various industrial applications, highlighting its efficiency and versatility in adsorption processes. The successful fabrication and optimization of the MW-AC makes it a valuable contribution to the field of environmental remediation and industrial pollution control.

## Data Availability

The data that support the current study are available from the corresponding author upon reasonable request.
